# MERS coronavirus: diagnostics, epidemiology and transmission

**DOI:** 10.1186/s12985-015-0439-5

**Published:** 2015-12-22

**Authors:** Ian M. Mackay, Katherine E. Arden

**Affiliations:** Department of Health, Public and Environmental Health Virology Laboratory, Forensic and Scientific Services, Archerfield, QLD Australia; The University of Queensland, St Lucia, QLD Australia; Queensland University of Technology, George St, Brisbane, QLD Australia

**Keywords:** Middle East respiratory syndrome, Coronavirus, MERS, Epidemiology, Diagnostics, Transmission

## Abstract

The first known cases of Middle East respiratory syndrome (MERS), associated with infection by a novel coronavirus (CoV), occurred in 2012 in Jordan but were reported retrospectively. The case first to be publicly reported was from Jeddah, in the Kingdom of Saudi Arabia (KSA). Since then, MERS-CoV sequences have been found in a bat and in many dromedary camels (DC). MERS-CoV is enzootic in DC across the Arabian Peninsula and in parts of Africa, causing mild upper respiratory tract illness in its camel reservoir and sporadic, but relatively rare human infections. Precisely how virus transmits to humans remains unknown but close and lengthy exposure appears to be a requirement. The KSA is the focal point of MERS, with the majority of human cases. In humans, MERS is mostly known as a lower respiratory tract (LRT) disease involving fever, cough, breathing difficulties and pneumonia that may progress to acute respiratory distress syndrome, multiorgan failure and death in 20 % to 40 % of those infected. However, MERS-CoV has also been detected in mild and influenza-like illnesses and in those with no signs or symptoms. Older males most obviously suffer severe disease and MERS patients often have comorbidities. Compared to severe acute respiratory syndrome (SARS), another sometimes- fatal zoonotic coronavirus disease that has since disappeared, MERS progresses more rapidly to respiratory failure and acute kidney injury (it also has an affinity for growth in kidney cells under laboratory conditions), is more frequently reported in patients with underlying disease and is more often fatal. Most human cases of MERS have been linked to lapses in infection prevention and control (IPC) in healthcare settings, with approximately 20 % of all virus detections reported among healthcare workers (HCWs) and higher exposures in those with occupations that bring them into close contact with camels. Sero-surveys have found widespread evidence of past infection in adult camels and limited past exposure among humans. Sensitive, validated reverse transcriptase real-time polymerase chain reaction (RT-rtPCR)-based diagnostics have been available almost from the start of the emergence of MERS. While the basic virology of MERS-CoV has advanced over the past three years, understanding of the interplay between camel, environment, and human remains limited.

**Electronic supplementary material:**

The online version of this article (doi:10.1186/s12985-015-0439-5) contains supplementary material, which is available to authorized users.

## Background

**Fig. 1:**
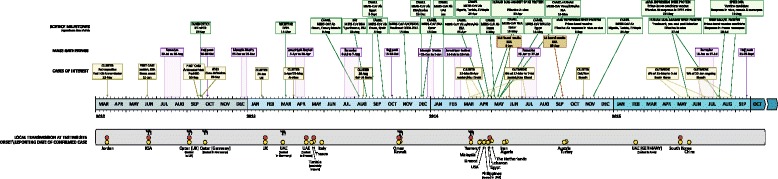
A timeline of some key scientific milestones, mass gatherings of relevance and clusters and outbreaks of interest to the understanding of MERS-CoV infection among humans and transmission from animals to humans. A yellow circle indicates when a country reported a laboratory confirmed detection and an orange circle denotes ensuing local transmission. A sample of the mentions of DC contact prior to disease is indicated by a black camel icon. DPP4-dipeptidyl peptidase 4; KSA-the Kingdom of Saudi Arabia; Mab-monoclonal antibody; rAdV-recombinant adenovirus; rMVA-recombinant modified vaccinia virus Ankara; UAE-United Arab Emirates

An email from Dr Ali Mohamed Zaki, an Egyptian virologist working at the Dr Soliman Fakeeh Hospital in Jeddah in the Kingdom of Saudi Arabia (KSA) announced the first culture of a new coronavirus to the world. The email was published on the website of the professional emerging diseases (ProMED) network on 20^th^September 2012 [1] (Fig. 1) and described the first reported case, a 60 year old man from Bisha in the KSA. This information led to the rapid discovery of a second case of the virus, this time in an ill patient in the United Kingdom, who had been transferred from Qatar for care [2]. The new virus was initially called novel coronavirus (nCoV) and subsequentlty entitled the Middle East respiratoy syndrome coronavirus (MERS-CoV). As of 2^nd^ of September 2015, there have been 1,493 detections of viral RNA or virus-specific antibodies across 26 countries (Additional file 1: Figure S1) confirmed by the World Health Organization (WHO), with over a third of the positive people dying (at least 527, 35 %) [3].

Since that first report, a slow discovery process over the following two to three years revealed a virus that had infected over 90 % of adult dromedary camels (DC; *Camelus dromedarius*) in the KSA [4], also DCs across the Arabian Peninsula and parts of Africa that are a source of DC imports for the KSA [5]. To date, MERS-CoV has not been detected in DCs tested in zoos or herds from other parts of the world [6–9]. Occasionally, virus is transmitted from infected DCs to exposed humans. Subsequent transmission to other humans requires relatively close and prolonged exposure [10].

The first viral isolate was patented and concerns were raised that this would restrict access to both the virus and to viral diagnostics [11, 12]. However, sensitive, validated reverse transcriptase real-time polymerase chain reaction (RT-rtPCR)-based diagnostics were quickly described and virus was made freely available subject to routine biosafety considerations [13]. Subsequent epidemiology and research has identified the cell receptor as exopeptidase dipeptidyl peptidase 4 (DPP4; also called CD26); that MERS-CoV has a broad tropism, replicating better in some cells lines and eliciting a more proinflammatory response than SARS-CoV; is widespread in DCs; has the potential to infect other animals and that MERS kills its human host more often than SARS did (20-40 % versus 9 % for SARS [14]) [15–19].

In humans, overt disease was given the name Middle East respiratory syndrome, with the acronym MERS. From intermittent animal-to-human spill-over events, the MERS-CoV spreads sporadically among people, causing more severe disease among older adults, especially males, with pre-existing diseases. The spread of MERS-CoV among humans has often been associated with outbreaks in hospitals, with around 20 % of all cases to date involving healthcare workers (HCWs).

## The Middle East Respiratory Syndrome (MERS)

Although DCs appear to suffer the equivalent of a ‘common cold’ from MERS-CoV infection, in humans, the virus can be a more serious and opportunistic pathogen associated with the death of up to 40 % of reported cases. It has yet to be established whether infections thought to have been acquired from an animal source produce a more severe outcome than those spread between humans [20]. Studies have established that the mean incubation period for MERS is five to six days, ranging from two to 16 days, with 13 to 14 days between when illness begins in one person and subsequently spreads to another [21–24]. Among those with progressive illness, the median time to death is 11 to 13 days, ranging from five to 27 days [23, 24]. Fever and gastrointestinal symptoms may form a prodrome, after which symptoms decline, only to be followed by a more severe systemic and respiratory syndrome [25, 26].

### The definition of a case

The first WHO case definition [27] defined probable cases of MERS based on the presence of febrile illness, cough and requirement for hospitalization with suspicion of lower respiratory tract (LRT) involvement. It also included roles for contact with a probable or confirmed case or for travel or residence within the Arabian Peninsula. If strictly adhered to, only the severe syndrome would be subject to laboratory testing, which was the paradigm early on [21]. From July 2013, the revised WHO case definition included the importance of seeking out and understanding the role of asymptomatic cases and from June 2014, the WHO definition more clearly stated that a confirmed case included any person whose sample was RT-PCR positive for MERS-CoV, or who produced a seroconversion, irrespective of clinical signs and symptoms. [28–30] Apart from the WHO and the KSA Ministry of Health reports, asymptomatic or subclinical cases of MERS-CoV infection were documented in the scientific literature although not always as often as occurred early on [31, 32]. The KSA definition of a case became more strict on 13^th^ May 2014, relying on the presence of both clinical features and laboratory confirmation [33]. Testing of asymptomatic people was recommended against from December 2014 [34], reinforced by a case definition released by the KSA Ministry of Health in June 2015 [35].

The KSA has been the source of 79 % of human cases. Severe MERS is notable for its impact among older men with comorbid diseases including diabetes mellitus, cirrhosis and various lung, renal and cardiac conditions [36–38]. Interestingly in June 2015, an outbreak in South Korea followed a similar distribution [39, 40]. Among laboratory confirmed cases, fever, cough and upper respiratory tract (URT) signs and symptoms usually occur first, followed within a week by progressive LRT distress and lymphopaenia [37]. Patients often present to a hospital with pneumonia, or worse, and secondary bacterial infections have been reported [37, 41]. Disease can progress to acute respiratory distress syndrome and multiorgan system failure [37]. MERS has reportedly killed approximately 35 % of all reported cases, 42 % of cases in the KSA, yet only 19 % of cases in South Korea, where mortality ranged from 7 % among younger age groups to 40 % among those aged 60 years and above [42]; all may be inflated values with asymptomatic or mild infections sometimes not sought or not reported [34]. General supportive care is key to managing severe cases [43]. Children under the age of 14 years are rarely reported to be positive for MERS-CoV, comprising only 1.1 % (n = 16) of total reported cases. Between 1^st^ September 2012 and 2^nd^ December 2013, a study described the then tally of paediatric cases in the KSA, which stood at 11 (two to 16 years of age; median 13 years); nine were asymptomatic (72 %) and one infant died [44]. In Amman, Jordan, 1,005 samples from hospitalized children under the age of two years with fever and/or respiratory signs and symptoms were tested but none were positive for MERS-CoV RNA, despite being collected at a similar time to the first known outbreak of MERS-CoV in the neighbouring town of Al-Zarqa [45]. A second trimester stillbirth occurred in a pregnant woman during an acute respiratory illness and while not RT-rtPCR positive, the mother did subsequently develop antibodies to MERS-CoV, suggestive of recent infection [46]. Her exposure history to a MERS-CoV RT-rtPCR positive relative and an antibody-reactive husband, her incubation period and her symptom history met the WHO criteria for being a probable MERS-CoV case [46].

## Laboratory testing to confirm past or present MERS-CoV infection

**Fig. 2:**
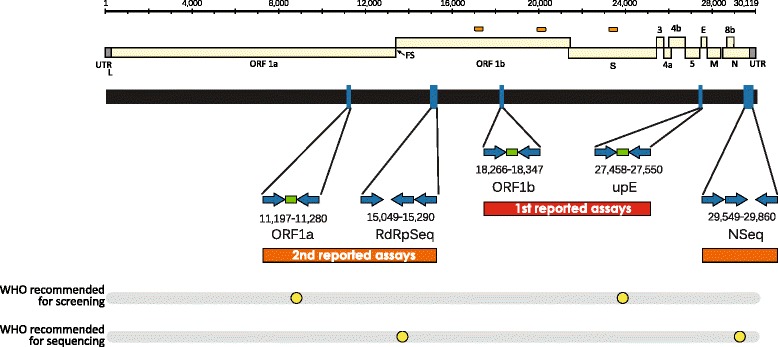
Schematic of MERS-CoV genome drawn to scale (EMC/2012; JX869059 [18].). Open reading frames are indicated as yellow rectangles bracketed by terminal untranslated regions (UTR; *grey rectangles*). FS-frame-shift. Predicted regions encompassing recombination break-points are indicated by orange pills. Created using Geneious v8.1 [211] and annotated using Adobe Illustrator. Beneath this is a schematic depicting the location of RT-PCR primers (*blue arrows* indicate direction) and oligoprobes (*green rectangles*) used in the earliest RT-rtPCR screening assays and conventional, semi-nested (three primers) RT-PCR confirmatory sequencing assays [47, 48]. Publication order is noted by first [27^th^ September 2012; red] and second [6^th^ December 2012; orange] coloured rectangles; both from Corman et al. [47, 48] Those assays recommended by the WHO are highlighted underneath by yellow dots [53]. The NSeq reverse primer has consistently contained one sequence mismatch with some MERS-CoV variants. An altered version of that from Mackay IM, Arden KE. Middle East respiratory syndrome: An emerging coronavirus infection tracked by the crowd. Virus Res 2015 Vol 202:60–88 with permission from Elsevier [5]

Diagnostic methods were published within days of the ProMED email announcing the first MERS case [47], including several now gold standard in-house RT-rtPCR assays (Fig. 2) as well as virus culture in Vero and LLC-MK2 cells [18, 47, 48]. A colorectal adenocarcinoma (Caco-2) epithelial cell line has since been recommended for isolation of infections MERS-CoV [49]. We previously reviewed the broad tropism of MERS-CoV [5]. However, as is well described, cell culture is a slow, specialised and insensitive method [50] while PCR-based techniques are the preferred method for MERS-CoV detection.

### Molecular detection of MERS-CoV RNA in real time

The first open reading frames (ORF 1a and 1b; Fig. 2) have become a key diagnostic and taxonomic target for CoV species identification. With less than 80 % identity between the amino acid sequence of MERS ORF 1ab and betacoronavirus relatives, *Tylonycteris* bat HKU4 and *Pipistrellus* bat HKU5, it can be concluded that it is a novel and distinct virus. MERS-CoV is predicted to encode ten open reading frames with 5’ and 3’ untranslated regions [51]. The structural proteins include the spike (S), envelope (E), membrane (M) and nucleocapsid (N) [52]. The products of ORF1a and ORF1b are predicted to encode nonstructural proteins.

The majority of specimen testing to date has employed validated RT-rtPCR assays shown to be sensitive and specific [47, 48, 53]. The RealStar® kit uses these WHO-recommended assays [54]. The target sequences of these screening assays have not changed among genomes examined until at least mid-2015 (IMM observation). Other RT-rtPCR assays have been developed and validated for use as laboratory-based diagnostic tools [55–57]. Additionally, loop-mediated [58, 59] or recombinase polymerase [60] isothermal assays have been designed for field deployment.

### MERS-CoV antigen detection

The detection of MERS-CoV antigen has not been common to date but the combination of short turnaround time from test to result, high throughput and identification of viral proteins makes this an attractive option. Detection of viral proteins rather than viral RNA indicates the likely presence of infectious virus. The first rapid immunochromatographic tool described could detect recombinant MERS-CoV nucleocapsid protein from DC nasal swabs with 94 % sensitivity and 100 % specificity compared to RT-rtPCR [61]. A different approach used a monoclonal antibody-based capture ELISA targeting the MERS-CoV nucleocapsid protein with a sensitivity of 10^3^ TCID_50_ and 100 % specificity [62].

### Assays to identify a humoral response to prior MERS-CoV infection among humans

Demonstration of a seroconversion to a MERS-CoV infection meets the current WHO definition of a case so optimized and thoroughly validated sero-assays employed alongside good clinical histories are useful to both identify prior MERS-CoV infection and help support transmission studies. Because serology testing is, by its nature, retrospective, it is usual to detect a viral footprint, in the form of antibodies, in the absence of any signs or symptoms of disease and often in the absence of any viral RNA [63].

Strategic, widespread sero-surveys of humans using samples collected after 2012 are infrequent. Much of the Arabian Peninsula and all of the Horn of Africa lack baseline data describing the proportion of the community who may have been infected by a MERS-CoV. However, sero-surveys have had widespread use in elucidating the role of DCs as a transmission source for MERS-CoV. Because of the identity shared between DC and human MERS-CoV (see *Molecular epidemiology: using genomes to understand outbreaks*), serological assays for DC sero-surveys should be transferrable to human screening with minimal re-configuration. Also, no diagnostically relevant variation in neutralization activity have been found from among a range of circulating tested MERS-CoV isolates and sera, so whole virus or specific protein-based sero-assays should perform equivalently in detecting serological responses to the single MERS-CoV serotype [49]. The development of robust serological assays requires reliable panels of well-characterized animal or human sera, including those positive for antibodies specific to MERS-CoV, as well as to likely sources of cross-reaction [64]. Obtaining these materials was problematic and slowed the development and commercialization of antibody detection assays for human testing [64]. A number of commercial ELISA kits, immunofluorescent assays (IFA) kits, recombinant proteins and monoclonal antibodies have been released [31, 65–68]. Initially, conventional IFAs were used for human sero-surveys. These relied on MERS-CoV-infected cell culture as an antigen source, detecting the presence of human anti-MERS-CoV IgG, IgM or neutralizing antibodies in human samples [18, 48, 69]. No sign of MERS-CoV antibodies was found among 2,400 sera from patients visiting Hospital in Jeddah, from 2010 through 2012, prior to the description of MERS-CoV [18]. Nor did IFA methods detect any sign of prior MERS-CoV infection among a small sample of 130 healthy blood donors from another Hospital in Jeddah (collected between Jan and Dec 2012) [70]. Of 226 slaughterhouse workers, only eight (3.5 %) were positive by IFA, and those sera could not be confirmed by virus neutralization (NT) test. The study indicated that HCoV-HKU1 was a likely source of cross-reactive antigen in the whole virus IFA [70]. Whole virus MERS-CoV IFA also suffered from some cross-reactivity with convalescent SARS patient sera and this could not be resolved by an NT test which was also cross-reactive [71]. IFA using recombinant proteins instead of whole-virus IFA, has been shown to be a more specific tool [31]. Since asymptomatic zoonoses have been posited [72], an absence of antibodies to MERS-CoV among some humans who have regular and close contact with camels may reflect the rarity of actively infected animals at butcheries, a limited transmission risk associated with slaughtering DCs [70], a pre-existing cross-protective immune status or some other factor(s) resulting in a low risk of disease and concurrent seroconversion developing after exposure in this group. IFA using recombinant proteins instead.

Some sero-assays have bypassed the risks of working with infectious virus by creating transfected cells expressing recombinant portions of the MERS-CoV nucleocapsid and spike proteins [48, 73], or using a recombinant lentivirus expressing MERS-CoV spike protein and luciferase [74, 75]. A pseudo particle neutralization (ppNT) assay has seen widespread used in animal studies and was at least as sensitive as the traditional microneutralization (MNT) test. [10, 74, 76–78] Studies using small sample numbers and ppNT found no evidence of MERS-CoV neutralizing antibody in sera from 158 children with LRT infections between May 2010 and May 2011, 110 sera from 19 to 52 year old male blood donors and 300 self-identified animal workers from the Jazan Region of the KSA during 2012 [79, 80]. Similarly, a study of four herdsmen in contact with an infected DC herd in Al-Ahsa, eight people who had intermittent contact with the herd, 30 veterinary surgeons and support staff who were not exposed to the herd, three unprotected abattoir workers in Al-Ahsa and 146 controls who were not exposed to DCs in any professional role, found none with serological evidence of past MERS-CoV infection using the ppNT assay [10]. A delay in the neutralizing antibody response to MERS-CoV infection was associated with increased disease severity in South Korea cases with most responses detectable by week three of illness while others, even though disease was severe, did not respond for four or more weeks [81]. The implications for our ability to detect any response in mild or asymptomatic cases was not explored but may be a signifcant factor in understanding exposure in the wider community.

A Jordanian outbreak of acute LRT disease in a hospital in 2012 was retrospectively found to be associated with MERS-CoV infection, initially using RT-rtPCR, but subsequently, and on a larger scale, through positivity by ELISA and IFA or MNT test. [46, 82, 83] This outbreak predated the first case of MERS in the KSA. The ELISA used a recombinant nucleocapsid protein from the group 2 betacoronavirus bat-CoV HKU5 to identify antibodies against the equivalent cross-reactive MERS-CoV protein [71]. It was validated using 545 sera collected from people with prior HCoV-OC43, HCoV-229E, SARS-CoV, HCoV-NL63, HRV, HMPV or influenza A(H1N1) infections but was reportedly less specific than the recombinant IFA discussed above. It was still considered an applicable tool for screening large sample numbers [82]. A protein microarray expressing the S1 protein subunit has also been validated and widely used for DC testing [5, 84]. Detection of MERS-CoV infection using ELISA or S1 subunit protein microarray [84] is usually followed by confirmatory IFA and/or a plaque-reduction neutralization (PRNT) [69, 70, 85] or MNT test. [74, 85, 86] This confirmatory process aims toensure the antibodies detected are able to specifically neutralize the intended virus and are not more broadly reactive to other coronaviruses found in DCs (bovine CoV, BCoV) or humans (HCoV-OC43, HCoV-229E, HCoV-NL63, HCoV-HKU1, SARS-CoV). In the largest study of human sera, a tiered diagnostic process assigned both recombinant IFA and recombinant ELISA positive sera to ‘stage 1’ seropositivity. A stage 2 seropositive result additionally required a suitably titred PRNT result [87]. The study found 15 sera collected in 2012 to 2013 from 10,009 (0.2 %) people in 13 KSA provinces contained MERS-CoV antibodies, but significantly higher proportions in occurred in camel shepherds (two of 87; 2.3 %) and slaughterhouse workers (five of 140; 3.6 %) [87]. Contemporary surveys are needed.

MERS-CoV does not appear to be easily transmitted from DCs to humans, or perhaps it is [72], but generally does not trigger a detectable immune response if only mild disease or asymptomatic infection results. Serology assays are in need of further validation in this area so care is required when moving newly developed diagnostic serology algorithms from a research setting to one that informs public health decisions. This was reinforced when a false positive US case, purported to have been infected after a handshake and two face-to-face meetings, did not withstand further confirmatory analysis using a more specific, NT assay and was subsequently retracted [88, 89].

### Specimen types for RT-PCR and length of viral shedding

The WHO recommends sampling from the LRT for MERS-CoV RT-rtPCR testing, especially when sample collection is delayed by a week or more after onset of symptoms. [53] LRT samples are also best for attempting isolation of infectious virus, although the success of culture is reduced when disease persists [49]. Recommended sample types include bronchoalveolar lavage (BAL), tracheal/tracheobronchial aspirate, pleural fluid and sputum [53, 90]. Fresh samples yield better diagnostic results than refrigerated material [69] and if delays in testing of ≥72 h are likely, samples (except for blood) should be frozen at −70 °C [90]. If available, lung biopsy or autopsy tissues can also be tested [53]. The URT is a less invasive and more convenient sampling site however, and an oropharyngeal and throat swab or a nasopharyngeal aspirate/wash are recommended when URT sampling is to be conducted [90]. Paired sera, collected two to three weeks apart are preferable for serological testing while a single sample is suggested to be sufficient if collected two weeks after onset of disease or a single serum collected during the first 10–12 days if conducting RT-rtPCR [53, 90]. Human urine and stool have been found to contain MERS-CoV RNA 12 to 26 days after symptom onset [25, 69, 91] and are listed as samples that should be considered [53, 90]. In two cases that arrived in the Netherlands, urine was RT-rtPCR negative but faeces was weakly positive and sera were RT-rtPCR positive for five days or more [25]. The finding of MERS-CoV viral RNA in serum provides an avenue for retrospective PCR-based studies if respiratory samples are unavailable [83]. RNAaemia may also correlate with disease severity; signs of virus were cleared from the serum of a recovered patient, yet lingered until the death of another [92].

Clinically suspected MERS cases may return negative results by RT-rtPCR. Data have shown one or more negative URT samples may be contradicted by further URT sampling or the use of LRT samples, which is preferred [2, 43, 93]. Higher viral loads occur in the LRT compared to the URT. [22, 69, 88, 94] This fits with the observation that the majority of disease symptoms are reported to manifest as systemic and LRT disease [21]. However, on occasion, even LRT specimens from MERS cases may initially be negative, only to later become positive by RT-PCR [95]. This may be due to poor sampling when a cough is absent or non-productive or because the viral load is low [95]. Despite this both the largest human MERS-CoV studies [32, 96–98] and smaller ones [22, 25, 99], use samples from the URT. It is then noteworthy that one study reported an association between higher loads in the URT and worse clinical outcome including intensive care and death [94]. At writing, no human data exist to define whether the virus replicates solely or preferentially in the LRT or URT, or replicates in other human tissues in vivo although MERS-CoV RNA has been detected from both the URT and LRT in a macaque monkey model [100].The distribution of DPP4 in the human upper airways is also not well described.

Individual human case studies report long periods of viral shedding, sometimes intermittently and not necessarily linked to the presence of disease symptoms. [25, 69, 99, 101] In one instance, a HCW shed viral RNA for 42 days in the absence of disease [99]. It is an area of high priority to better understand whether such cases are able to infect others. Over three quarters of MERS cases shed viral RNA in their LRT specimens (tracheal aspirates and sputum) for at least 30 days, while only 30 % of contacts were still shedding RNA in their URT specimens [91, 102].

In the only study to examine the effect of sample type on molecular analysis, 64 nasopharyngeal aspirates (NPA; an URT sample), 30 tracheal aspirates, 13 sputa and three BAL were examined. The tracheal aspirates and BAL returned the highest viral load values followed by NPA and sputum. Unsurprisingly, higher viral loads generally paralleled whole genome sequencing and culture success and, in NPA testing, were significantly correlated with severe disease and death [49, 94, 103]. This study demonstrated the importance of LRT sampling for whole genome sequencing.

### MERS-CoV and concurrent infections

When tested, samples positive for MERS-CoV are often negative for other pathogens [2, 25, 93, 104]. However, many studies make no mention of additional testing for endemic human respiratory viruses [21, 23, 73, 105]. When viruses are sought, they have included human herpesvirus (HHV), rhinoviruses (HRV), enteroviruses (EV), respiratory syncytial virus (RSV), parainfluenzavirus types 1, 2 and 3 (PIVs),influenzaviruses (IFVs), endemic HCoVs, adenoviruses (AdVs) metapneumovirus (MPV) and influenza A\H1N1 virus; co-detections with MERS-CoV have been found on occasion [2, 22, 37, 69, 97]. Bacterial testing is sometimes included (for example, for *Legionella* and *Pneumococcus*) but the impact of bacterial co-presence is also unclear [22, 104–106]. Further testing of the LRT sample from the first MERS case used IFA to screen for some viruses (negative for IFV, PIVs, RSV and AdVs) and RT-PCR for others (negative for AdV, EVs, MPV and HHVs) [18]. RT-PCR also detected MERS-CoV. The WHO strongly recommends testing for other respiratory pathogens [53] but with this recommendation often discounted, there are limited data to address the occurrence and impact of co-infections or alternative viral diagnoses among both MERS cases and their contacts. Little is known of other causes of MERS-like pneumonia in the KSA or of the general burden of disease due to the known classical respiratory viruses.

## Mass MERS-CoV screening studies

Testing of adult pilgrims performing the Hajj in 2012 to 2014 has not detected any MERS-CoV. In 2012, nasal swabs from 154 pilgrims collected prior to leaving for or departing from the KSA were tested [47]. In 2013, testing was significantly scaled up with 5,235 nasopharyngeal swabs from 3,210 incoming pilgrims and 2,025 swabs from outgoing pilgrims tested [98]. It should be noted that most pilgrims arrived from MERS-free countries. A further 114 swabs were taken from pilgrims with influenza-like illness [96, 107]. In earlier Hajj gatherings, it was found that influenza viruses circulated widely, whilst other viruses, often rhinoviruses, circulated more selectively, interpreted as indicating their importation along with foreign pilgrims. [107–109] Over time, increased influenza vaccination has been credited for a fall in the prevalence of influenza like illnesses among Hajj pilgrims. [110] A LRT sample is often not collected for these studies [98, 107, 109], so false negative findings are a possibility although little is known about the initial site of MERS-CoV infection and replication; it may have been assumed it was the LRT because disease was first noticed there but the URT may be the site of the earliest replication.

**Fig. 3:**
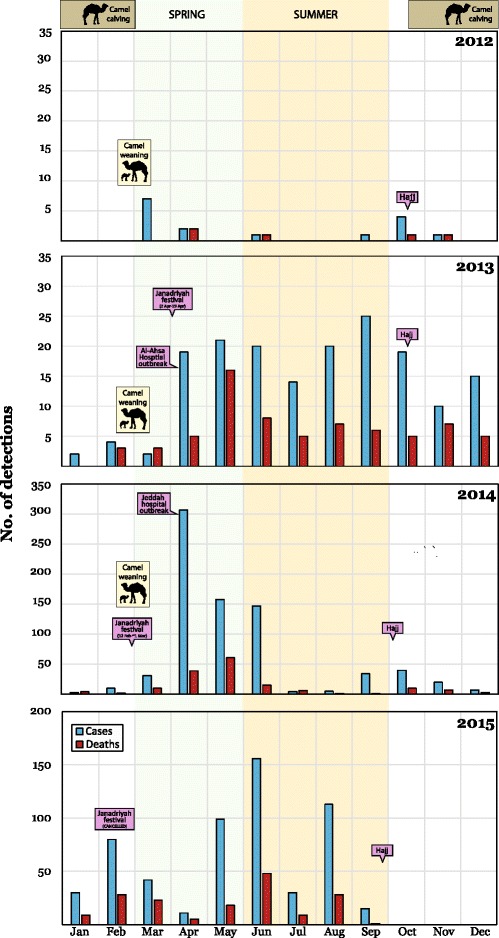
Monthly detections of MERS-CoV (*blue bars*) and of cases who died (*red bars*) with some dates of interest marked for 2012 to 4^th^ September 2015. An approximation of when DC calving season [128] and when recently born DCs are weaned is indicated. Spring (*green*) and summer (*orange*) in the Arabian Peninsula are also shaded. Note the left-hand y-axis scale for 2014 and 2015 which is greater than for 2012/13. Sources of these public data include the WHO, Ministries of Health and FluTrackers [207–209]. Earlier and subsequent versions of this chart are maintained on a personal blog [210]. Modified and reprinted from Mackay IM, Arden KE. Middle East respiratory syndrome: An emerging coronavirus infection tracked by the crowd. Virus Res 2015 Vol 202:60–88 with permission from Elsevier [5]

In Jeddah between March and July 2014 (hereafter called the Jeddah-2014 outbreak; Fig. 3), there was a rapid increase in MERS cases, accompanied by intense screening; approximately 5,000 samples from in and around the region were tested in a month yielding around 140 MERS-CoV detections (~3 % prevalence) [111]. Among 5,065 individuals sampled and tested across the KSA between October 2012 and September 2013,108 (2.1 %) detections were made in a hospital-centric population which included hospitalized cases (n = 2,908; 57.4 %), their families (n = 462; 9.1 %) and associated HCWs (n = 1,695; 33.5 %) [32]. Among the detections, 19 (17.8 %) were HCWs and 10 (9.3 %) were family contacts [32].

The 2-3 % prevalence of active MERS-CoV infections is not dissimilar to the hospital-based prevalence of other human CoVs. [112] However, the proportion of deaths among those infected with MERS-CoV is much higher than that known for the HCoVs NL63, HKU1, 229E or OC43 in other countries, and even above that for SARS-CoV; it is not a virus that could reasonably be described as a “storm in a teacup”. It is the low transmission rate that has prevented worldwide spread, despite many “opportunities”.

## Sporadic spill-over and facilitated outbreaks

**Fig. 4:**
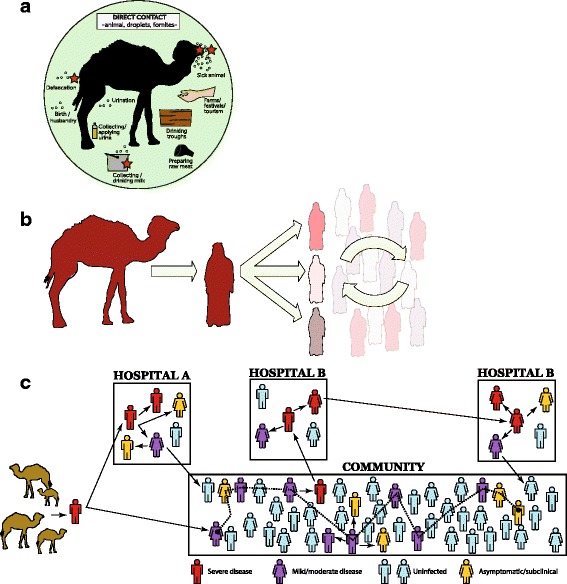
A speculative series of how humans and DCs contribute to the global tally of MERS cases. **a**. Risks for acquiring MERS-CoV from a DC. This illustration highlights risks that may originate from a droplet transmission component (be they larger, heavier wet droplets or the drier, airborne gel-like droplet nuclei) or a direct contact component (within the *green circle*). No routes of MERS-CoV acquisition to or between humans have been proven to date. Modified and reprinted from Mackay IM, Arden KE. Middle East respiratory syndrome: An emerging coronavirus infection tracked by the crowd. Virus Res 2015 Vol 202:60–88 with permission from Elsevier [5]. **b** Camel-to-human infections appear to be infrequent, while human-to-human spread of infection is regularly facilitated by poor IPC in healthcare settings where transmission is amplified, accounting for the bulk of cases. There are human MERS cases that do not fall into either category of source and it is unclear if these acquired infection through some entirely separate route, or from cases that escaped diagnosis. **c** Hypothetical ways in which subclinical (when infection may not meet a previously defined clinical threshold of signs and/or symptoms) or asymptomatic (no obvious signs or measured, noticed or recalled symptoms of illness) MERS-CoV infection may be implicated in transmission

Very early in the MERS outbreak, some animals were highly regarded as either the reservoir or intermediate host(s) of MERS-CoV with three of the first five cases having contact with DCs [73, 113, 114]. Today, animal MERS-CoV infections must be reported to the world organization for animal health as an emerging disease [115]. A summary of the first MERS cases reported by the WHO defined animal contact with humans as being direct and within 10 days prior to symptom onset [20]. This definition made no specific allowance for acquisition from DCs through a droplet-based route, which is very likely route for acquisition of a virus that initially and predominantly causes respiratory disease [23]. Camels are known to produce high levels of MERS-CoV RNA in their URT and lungs [116]. Providing support for a droplet transmission route and perhaps indicating the presence of RNA in smaller, drier droplet nuclei, MERS-CoV RNA was identified in a high volume air sample collected from a barn housing an infected DC [117]. The precise source from which humans acquire MERS-CoV remains poorly studied but it seems likely that animal and human behavioural factors may play roles (Fig. 3) [118]. These factors may prove important for human cases who do not describe any DC contact [119] nor any contact with a confirmed case. Whether the WHO definition of animal contact is sufficient to identify exposure to this respiratory virus remains unclear. Wording focuses on consumption of DC products but does not specifically ascribe risk to a droplet route for acquisition of MERS-CoV from DC [120]. Some MERS patients are listed in WHO disease notices as being in proximity to DCs or farms, but the individuals have not described coming into contact with the animals. No alternative path for acquiring infection is reported in many of these instances. What constitutes a definition of “contact” during these interviews has been defined for one study [72]. Despite this lack of clarity, the WHO consider that evidence linking MERS-CoV transmission between DCs to humans is irrefutable (Fig. 4) [120].

The possibility that bats were an animal host of MERS-CoV was initially widely discussed because of the existing diversity of coronaviruses known to reside among them [121–124]. Conclusive evidence supporting bats as a source for human infections by MERS-CoV has yet to be found, but bats do appear to host ancestral representatives [53, 125]. However, these are not variants of the same virus nor always within the same phylogenetic lineage as MERS-CoV; they are each a genetically distinct virus. Bat-to-human infection by MERS-CoV is a purely speculative event. The only piece of MERS-CoV-specific evidence pointing to bats originates from amplification of a 190 nt fragment of the RNA-dependent RNA polymerase gene of the MERS-CoV genome, identified in a faecal pellet from an insectivorous *Emballonuridae* bat, *Taphozous perforatus* found in Bisha, the KSA [121]. While very short, the sequence of the fragment defined it as a diagnostic discovery. Subsequently a link to DCs was reported [85] and that link has matured into a verified association [38, 126] (Fig. 4).

DCs, which make up 95 % of all camels, have a central presence in the Arabian Peninsula where human-DC contact ranges from little to close [119]. Contact may be commonplace and could occur in variety of ways (Fig. 4a). There are several large well-attended festivals, races, sales and parades which feature DCs and DCs are also kept and bred close to populated areas in the KSA [127, 128]. DC milk and meat are widely consumed and the older DC is an animal of ritual significance after the Hajj pilgrimage [129]. However, MERS-CoV infection frequency is reportedly much lower than is the widespread and frequent habit of eating, drinking and preparing DC products. Daily ingestion of fresh unpasteurized DC milk is common among the desert Bedouin and many others in the KSA. DC urine is also consumed or used for supposed health benefits. Despite camel butchery being a local occupation, neither butchers nor other at-risk groups are identifiable among MERS cases; this may simply be a reporting issue rather than an unexplainable absence of MERS. A small case–control study published in 2015 identified direct DC contact, and not ingestion of products, to be associated with onset of MERS [38].

The first sero-survey of livestock living in the Middle East region was conducted during 2012–2013 [85]. DCs were sampled from a mostly Canary Island-born herd and from Omani DCs (originally imported from the Horn of Africa) [85]. A neutralising antibody assay found only 10 % of strongly seropositive Canary Island DC sera could neutralise MERS-CoV while all Omani DC sera had high levels of specific MERS-CoV neutralizing antibody [85]. This indicated that DCs had in the past been infected by MERS-CoV, or a very similar virus.

Since this study, a host of peer-reviewed reports have looked at both DCs and other animals, and the possibility that they may host MERS-CoV infection. Seropositive DCs have been found throughout the Arabian Peninsula including Oman, the KSA, Qatar, Jordan, the United Arab Emirates (UAE), Kuwait as well as Sudan, Somalia, Egypt, Tunisia, Nigeria, Kenya and Ethiopia in Africa and the Canary Islands [85, 130–134]. Other animals tested include sheep, cows, pigs, horses, donkeys, mules, birds, water buffalo, goats, Bactrian camels, llamas and guanaco (south American camelids) but none had detectable neutralising antibody against MERS-CoV [4, 74, 78, 85, 86, 135, 136]. No virology or serology studies of human samples from areas in Africa where there are camels with a history of MERS-CoV have been reported to date. However,an absence of unexplained pneumonia that may be attributable to MERS-CoV infection may not signal the absence of virus among humans in each country but simply reflect a lack of expensive epidemiology studies conducted by resource-poor countries.﻿ It is thus unclear whether MERS-CoV, or an antigenically related CoV, is an unrecognized pathogen in these regions, perhaps circulating for even longer than it has been known in the Arabian Peninsula [133].

MERS-CoV RNA has also been detected in DC samples, and recovery of infectious virus has also been achieved from DC samples [4, 77, 117, 132, 137–141]. From some of these, full or majority length genomes of MERS-CoV have been sequenced [77, 137, 138]. DC versions of MERS-CoV were found to be as similar to each other, as were variants detected from different humans over time and across distance.

Antibody screening assays have also detected cross-reactive antibodies in sera. These were identified as such by screening sera against similar viruses, for example BCoV or HCoV-OC43 (as an antigenic facsimile for BCoV). It is possible that other MERS-CoV-like viruses also reside within DCs, but this does not detract from the definitive finding of MERS-CoV genetic sequences in both DCs and humans [117, 142, 143].

Screening studies have shown that juvenile DCs are more often positive for virus or viral RNA while older DCs are more likely to be seropositive and RNA or virus negative [76, 77, 144]. In adult DCs, MERS-CoV RNA has been detected among animals with pre-existing antibody, suggesting re-infection is possible [77, 144]. Viral loads among positive DCs can be very high [4, 76, 77, 139, 144] and DCs have been found positive both when ill with URT respiratory signs [77, 117, 142, 145] or when apparently healthy [137]. These findings indicate DCs host natural MERS-CoV infections. Furthermore, stored DC sera have revealed signs of MERS-CoV in DCs which date back over three decades (the earliest collected in 1983) [4, 133, 135]. Older sera have not been tested and so precisely how long DCs have been afflicted by MERS-CoV, whether the virus is enzootic among them, introduced to them decades or centuries ago from bats in Africa or the Arabian Peninsula, or they are the subject of regular but short-lived viral incursions from an as yet unknown host, cannot be answered.

Researchers sought to determine a direction for infection; were DCs transmitting virus to humans or were humans infecting DCs? At a Qatari site, a farm owner and his employee became ill in mid-October 2013 and tested positive for MERS-CoV RNA in a sputum and throat swab sample, respectively. RT-rtPCRs found MERS-CoV RNA in 11 of 14 positive DC nasal swabs at the farm; six (43 %) positive by two or more assays [138]. The results indicated a recent outbreak had occurred in this herd; the first indication of MERS-CoV RNA found within DCs with a temporal association to human infections. Three positive DC samples were confirmed by sequencing a 358 nt portion of the spike gene; these sequences were identical to each other, again with close homology to other human and DC MERS-CoV sequences [138]. The DCs and human contacts yielded ORF1a and ORF4b sequences differing by only a single nucleotide each, clustering closely with the Hafr-Al-Batin_1_2013 variant [138]. Subsequent case studies found evidence of a concurrent human and DC infection and the direction of that infection was inferred to be from the ill DCs and to their human owners [117, 142, 146]. Partial genome sequences indicated that a human and a MERS-CoV RT-rtPCR positive DC had been infected by a variant of the same virus, harbouring the same distinct pattern of nucleotide polymorphisms. [142] All nine DC in the owner’s herd, serially sampled, reacted in a recombinant S1 antigen ELISA, with the two animals that had been RT-rtPCR positive showing a small, verifiable rise in antibody titre [142]. A rise in titre theoretically begins 10 to 21 days after DC infection [142]. The authors suggested that the rise in titre in DC sera which occurred alongside a declining RNA load, while the patient was actively ill and hospitalized, indicated that the DCs were infected first followed by the owner [117, 142]. BCoV antibodies were also present, and rising in one of the two RT-rtPCR positive animals but no animal’s antibodies could neutralise BCoV infection [142].

Camel calving season occurs in the winter months (between late October and late February; Fig. 3) and this may be a time when there is increased risk to humans of spill-over due to new infections among naïve DC populations [128]. What role maternal camel antibody might play in delaying infection of calves remains unknown [128, 142]. Juvenile DCs appear to host active infection more often than adult DCs and thus the sacrificial slaughter of DCs, which must be five years of age or older (termed a thane), may not be accompanied by significant risk of exposure to infection. In contrast to earlier results, slaughterhouse workers who kill both younger and older DCs, may be an occupational group with significantly higher incidence of seropositivity to MERS-CoV when animals have active MERS-CoV infections [129, 139, 147–149]. Expanded virological investigations of African DCs may lead to more seropositive animals and geographic areas in which humans may be at risk. It is possible that there are areas where humans already harbour MERS-CoV infections that have not been identified because of an absence of laboratory surveillance. Virological investigations of bats may lead to findings of ancestral viruses and viral 'missing links' and identifying any other animal sources of zoonotic spread is important to inform options for reducing human exposures [56, 76].

### Virus survival in the environment

Infectious MERS-CoV added to DC, goat or cow milk and stored at 4 °C could be recovered at least 72 h later and, if stored at 22 °C, recovery was possible for up to 48 h [150]. MERS-CoV titre decreased somewhat when recovered from milk at 22 °C but pasteurization completely ablated MERS-CoV infectivity [150]. In a subsequent study, MERS-CoV RNA was identified in the milk, nasal secretion and faeces of DCs from Qatar [151].

A single study has examined the ability of MERS-CoV to survive in the environment [150]. Plastic or steel surfaces were inoculated with 10^6^ TCID_50_ of MERS-CoV at different temperature and relative humidity (RH) and virus recovery was attempted in cell culture. At high ambient temperature (30 °C) and low RH (30 %) MERS-CoV remained viable for 24 h [150]. By comparison, a well known and efficently transmitted respiratory virus, influenza A virus, could not be recovered in culture beyond four hours under any conditions [150]. Aerosol experiments found MERS-CoV viability only decreased 7 % at low RH at 20 °C. In comparison, influenza A virus decreased by 95 % [150]. MERS-CoV survival is inferior to that previously demonstrated for SARS-CoV [152]. For context, pathogenic bacteria can remain viable and airborne for 45 min in a coughed aerosol and can spread 4 m. MERS-CoV’s ability to remain viable over long time periods gives it the capacity to thoroughly contaminate a room’s surfaces when occupied by an infected and symptomatic patient [153]. Whether MERS-CoV can remain adrift and infectious for extended periods (truly airborne) remains unknown. Such findings expand our understanding of the possibilities for droplets to transmit respiratory viruses in many settings, including hospital waiting rooms, emergency departments, treatment rooms, open intensive care facilities and private patient rooms. The nature and quality of air exchange, circulation and filtration are important variables in risk measurement and reduction as is the use of negative pressure rooms to contain known cases. Droplet spread between humans is considered the mechanism of human-to-human transmission and the need for droplet precautions was emphasized after the Al-Ahsa hospital, the KSA and the South Korean outbreaks [21, 23, 154, 155]. By extrapolation, aerosol-generating events involving DCs (urination, defecation, and preparation and consumption of DC products) should be factored into risk measurement and reduction efforts and messaged using appropriate context. The provision of evidence supporting the best formulation of personal protective equipment to be worn by HCWs who receive, manage or conduct procedures on infectious cases remains a priority.

### Transmission of MERS-CoV among humans

MERS-CoV was found and characterized because of its apparent association with severe, and therefore more obvious, illness in humans; we were the canaries in the coal mine. Sero-assays and prospective cohort studies have yet to determine the extent to which milder or asymptomatic cases contribute to MERS-CoV transmission chains. However, transmission of MERS-CoV is defined as sporadic (not sustained), intra-familial, often healthcare associated, inefficient and requiring close and prolonged contact [22, 31, 63, 93, 97, 102, 156] In a household study, 14 of 280 (5 %) contacts of 26 MERS-CoV positive index patients were RNA or antibody positive; the rate of general transmission, even in outbreaks is around 3 % [31]. It seems that the majority of human cases of MERS-CoV, even when numbers appear to increase suddenly, do not readily transmit to more than one other human so to date, the localized epidemic of MERS-CoV has not been self-sustaining [157–161]. That is to say, the basic reproduction number (R_0_) - the average number of infections caused by one infected individual in a fully susceptible population – has been close to one throughout various clusters and outbreaks. If R_0_ was greater than 1, a sustained increase in case numbers would be expected. Some R_o_ calculations may be affected by incomplete case contact tracing, limited community testing and how a case is defined. That MERS has had a constant presence in the Arabian Peninsula since 2012 is due to ongoing, sporadic spill-over events from DCs amplified by poorly controlled hospital outbreaks.

The first known MERS human-to-human transmission event was one characterized by acute LRT disease in a healthcare setting in Jordan. In stark contrast, a sero-survey of HCW who were sometimes in close and prolonged contact with the first, fatal MERS-CoV case in 2012 [162], found none of the HCW had seroconverted four months later, despite an absence of eye protection and variable compliance with required PPE standards [162].

Early on in the MERS story, samples for testing were mostly collected from patients with severe illness and not those with milder acute respiratory tract infections. Contacts of confirmed MERS cases were often observed for clinical illness, but not tested. These omissions may have confounded our understanding of MERS-CoV transmission and biased early data towards higher numbers of seriously ill and hospitalized patients, inflating the apparent proportion of fatal cases. Case–control studies were not a focus. As testing paradigms changed and contacts were increasingly tested, more asymptomatic and mild infections were recognized [163].

A rise in the cases termed asymptomatic (which enlarge the denominator for calculations of the proportion of fatal cases, defined in [164]) resulted in a drop in the proportion of fatal cases during the Jeddah-2014 outbreak. Historically, such rises are consistent with changing definitions and laboratory responses and clinical management of a newly discovered virus infection that was first noted only among the severely ill. Upon follow-up, over three-quarters of such MERS-CoV RNA positive people did recall having one or more symptoms at the time, despite being reported as asymptomatic [165] raising some question over the reliability of other reported data.

The proportion of fatal MERS cases within the KSA compared to outside the KSA, as well as the age, and sex distribution change in different ways when comparing MERS outbreaks. Approximately 43 % of MERS cases (549 of 1277) in the KSA were fatal betwen 2012 and December 2015 while 21 % (72 of 330) died among those occurring outside of the KSA. The total number of male cases always outnumber females and the proportion of male deaths is always greater than the proportion of females who die. However the proportion of male deaths from total males with MERS is a similar figure to that for females. In the KSA, there is a greater proportion of younger males among cases and deaths than were observed from the 2015 South Korean or the Jeddah-2014 outbreaks (Additional file 2: Figure S2). Why these aspects have differed may be due to differences in the time to presentation and diagnosis, the nature and quality of supportive care, the way a person became infected (habits, exposure to a human or zoonotic source, viral load, route of infection) or the extent to which different populations are burdened by underlying diseases [40].

**Fig. 5:**
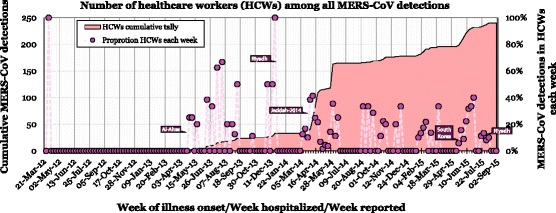
Data on MERS-CoV detections among HCWs based on publicly described laboratory confirmed cases collated into the author’s curated line list as at 4^th^ September 2015. Sources of these public data include the WHO, Ministries of Health and FluTrackers [207–209]. Earlier and subsequent versions of this chart are maintained on a personal blog [210]

As a group, HCWs comprised 16 % of MERS cases in the KSA and South Korea. It is apparent that the weekly proportion of infected HCWs increases alongside each steep rise in overall detections (Fig. 5). In May 2013, the WHO published guidelines for IPC during care of probable or confirmed cases of MERS-CoV infection in a healthcare setting [166]. This is explainable because to date, each case rise has been intimately associated with healthcare-facility related outbreaks [118]. These rises in MERS-CoV detections can decrease the average age during each event because HCWs are usually younger than inpatients with MERS. Healthcare facilities have been a regular target for suggested improvements aimed at improving infection prevention and control (IPC) procedures [115, 118].

### Molecular epidemiology: using genomes to understand outbreaks

**Fig. 6:**
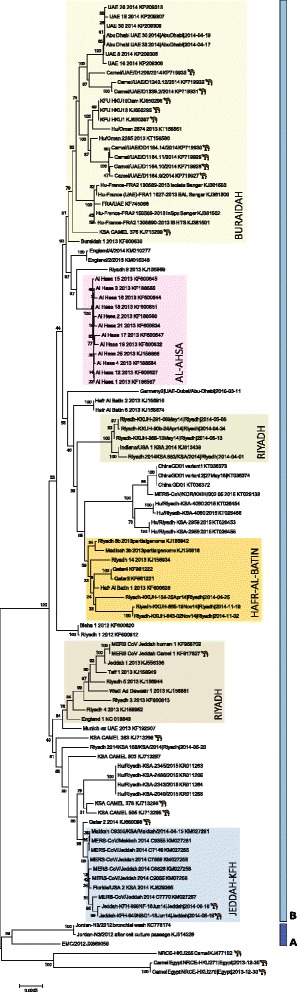
The genetic relationship between MERS-CoV nucleotide sequences (downloaded from GenBank using the listed accession numbers and from virological.org [212]). This neighbour joining tree was created in MEGA v6 using an alignment of human and DC-derived MERS-CoV sequences (Geneious v8.1 [211]). Clades are indicated next to dark (Clade A) or pale (Clade B) blue vertical bars. Camel icons denote genomes from DCs. Healthcare or community outbreaks are boxed and labelled using previously described schemes [212, 213]

Most of the analysis of MERS-CoV genetics has been performed using high throughput or “deep” sequencing methods for complete genome deduction [167–169]. MERS-CoV was the first subject of such widespread use of deep sequencing to study an emerging viral outbreak with global reach. The technique can produce genomic length coverage in a single experiment with highly repetitious measurement of each nucleotide position [52, 140]. Despite assays having been published early on, subgenomic sequencing, once the mainstay of viral outbreak studies, has less often been published during MERS-CoV characterization [48]. As more genomes from both humans and DCs have been characterized, two clades have become apparent; A and B (Fig. 6). Clade A contains only human-derived MERS-CoV genomes from Jordan, while Clade B comprises the majority of human and camel genomes deduced thus far [168].

Two studies during 2015, one looking at Jeddah-2014 MERS-CoV variants and another looking at a variant exported from South Korea to China, have now identified signs of genetic recombination among MERS-CoV variants. While human and camel whole genome sequences have retained >99 % identity with each other, members of genetically distinct lineages can and do swap genetic material when suitable conditions and coinfections co-occur [170–172]. Shared identity implies that the major source for human acquisition is the DC, rather than another animal, although more testing of other animal species is needed to confirm that conclusion. Over a month, a DC virus sequenced on different occasions did not change at all indicating a degree of genomic stability in its host, supporting that DCs are the natural, rather than intermediate, host for the MERS-CoV we know today [77]. To date, recombination has been localised to breakpoints near the boundary between ORF1a and ORF1b regions, within the spike gene [170] and in the ORF1b region (Fig. 2) [172]. It is not unexpected that recombination should occur since it is well known among other CoVs [124] and because the majority of MERS-CoV whole genomes collected from samples spanning three years (2012–2015) and from humans, camels and different countries have shown close genetic identity to each other, with just enough subtle variation to support outbreak investigations so long as whole genome sequencing is applied [52, 77, 135, 138, 168, 173–175].

Changes in genome sequence may herald alterations to virus transmissibility, replication, persistence, lethality or response to future drugs. If we have prior knowledge of the impact of genetic changes because of thorough characterization studies, we can closely monitor the genomic regions and better understand any changes in transmission or disease patterns as they occur. Genetic mutations noted during the largest of human outbreaks, Jeddah-2014, did not impart any major replicative or immunomodulatory changes when compared to earlier viral variants in vitro [156, 176]. However, we understand very little of the phenotypic outcomes that result from subtle genetic change in MERS-CoV genomes. To date no clinical relevance or obvious in vivo changes to viral replication, shedding or transmission has been reported or attributed to mutations or to new recombinant viruses [156]. But vigilance and larger, more contemporary and in vivo studies are needed.

Genome sequence located to a distinct clade were identified from an Egyptian DC that was probably imported from Sudan. This does not fit into either of the current clades [125, 168, 177]. A virus sequenced from a *Neoromicia capensis* bat was more closely related to MERS-CoV than other large bat-derived sequences had been to that point, but the genome of a variant of a MERS-CoV has yet to be discovered and deduced from any bat [125].

Analyses of MERS-CoV genomes have shown that most single nucleotide differences among variants were located in the last third of the genome (Fig. 2), which encodes the spike protein and accessory proteins [168]. At least nine MERS-CoV genomes contained amino acid substitutions in the receptor binding domain (RBD) of the spike protein and codons 158 (N-terminal region), 460 (RBD), 1020 (in heptad repeat 1), 1202 and 1208 bear investigation as markers of adaptive change [140, 169]. The spike protein had not changed in the recombinant MERS-CoV genome identified in China in 2015 but was reported to have varied at a higher rate than that for complete MERS-CoV genomes, among South Korean variants [172, 178]. This highlights that subgenomic regions may not always contain enough genetic diversity to prove useful for differentiating viral variants. Despite this, one assay amplifying a 615 nucleotide fragment of the spike S2 domain gene for Sanger sequencing agreed with the results generated by the sequencing of a some full genomes and was useful to define additional sequence groupings [177].

Genomic sequence can also be used to define the geographic boundaries of a cluster or outbreak and monitor its progress, based on the similarity of the variants found among infected humans and animals when occurring together, or between different sites and times (Fig. 6) [169]. This approach was employed when defining the geographically constrained MERS hospital outbreak in Al-Ahsa, which occurred between 1^st^ April and 23^rd^ May 2013, as well as clusters in Buraidah and a community outbreak in Hafr Al-Batin, the KSA. Genomic sequencing identified that approximately 12 MERS-CoV detections from a community outbreak in Hafr Al-Batin between June and August 2013 may have been triggered by an index case becoming infected through DC contact [175]. Sequencing MERS-CoV genomes from the 2013 Al-Ahsa hospital outbreak indicated that multiple viral variants contributed to the cases but that most were similar enough to each other to be consistent with human-to-human transmission. Molecular epidemiology has revealed otherwise hidden links in transmission chains encompassing a period of up to five months [179]. However, most outbreaks have not continued for longer than two to three months and so opportunities for the virus to adapt further to humans through co-infection and sustained serial passage have been rare [169]. In Riyadh-2014, genetic evidence supported the likelihood of multiple external introductions of virus, implicating a range of healthcare facilities in an event that otherwise looked contiguous [23, 168, 179]. Riyadh is a nexus for camel and human travel and has had more MERS cases than any other region of the KSA to date but also harbours a wide range of MERS-CoV variants [128, 167, 179]. However the South Korean outbreak originated from a single infected person, resulting in three to four generations of cases [180, 181]. Studies of this apparently recombinant viral variant did not find an increased evolutionary rate and no sign of virus adaptation thus the outbreak seems to have been driven by circumstance rather than circumstance together with mutation [181].

### Contact tracing and the possible importance of asymptomatic cases

For many MERS cases detected outside the Arabian Peninsula, extensive contact tracing has been performed and the results described in detail. Contact tracing is essential to contain the emergence and transmission of a new virus and today it is supported by molecular epidemiology. Although it is an expensive and time consuming process, contact tracing can identify potential new infections and through active or passive monitoring, react more rapidly if disease does develop. Results of contact tracing to date have found that onward transmission among humans is an infrequent event. For example, there were 83 contacts, both symptomatic and asymptomatic, of a case treated in Germany who travelled from the UAE but no sign of virus or antibody were found in any of them [73]. The very first MERS case had made contact with 56 HCWs and 48 others, but none developed any indication of infection [162]. In a study of 123 contacts of a case treated in France, only seven matched the definition for a possible case and were tested; one who had shared a 20 m^2^ hospital room while in a bed 1.5 m away from the index case for a prolonged period was positive [26]. None of the contacts of the first two MERS cases imported into the USA in 2014 contained any MERS-CoV footprint [182] and none of the 131 contacts of two travellers returning to the Netherlands developed MERS-CoV antibodies or tested RNA positive [25, 183]. Analyses of public data reveal many likely instances of nosocomial acquisition of infection in the Arabian Peninsula and these data may be accompanied by some details noting contact with a known case or facility. One example identified the likely role of a patient with a subclinical infection, present in a hospital during their admission for other reasons, as the likeliest index case triggering a family cluster [93]. Contact tracing was a significant factor in the termination of a 2015 outbreak involving multiple South Korean hospitals [184]. Such studies demonstrate the necessity of finding and understanding a role for mild and asymptomatic cases, together with restricting close contact or prolonged exposure of infected people to others, especially older family members and friends with underlying disease (Fig. 4c).

### Hospital associated MERS outbreaks

The hospital-associated outbreak in Jeddah in 2014 was the largest and most rapid accumulation of MERS-CoV detections to date. The greatest number of MERS-CoV detections of any month on record occurred in Jeddah in April. The outbreak was mostly (>60 % of cases) associated with human-to-human spread within hospital environments and resulted from a lack of, or breakdown in, infection prevention and control [37, 185, 186]. A rise in fatalities followed the rapid increase in case numbers.

In 2015 two large outbreaks occurred. South Korea was the site of the first large scale outbreak outside the Arabian Peninsula and produced the first cases in both South Korea and China, occurring between May and July 2015. This was closely followed by a distinct outbreak in Ar Riyad province in the KSA which appeared to come under control in early November.

After staying in Bahrain for two weeks, a 68 year old male (68 M) travelled home to South Korea via Qatar, arriving free of symptoms on the 4^th^ May 2015 [187]. He developed fever, myalgia and a cough nearly a week later (11^th^). He visited a clinic as an outpatient between the 12^th^ and 15^th^ of May and was admitted to Hospital A on the 15^th^ [188]. He was discharged from Hospital A on the 17^th^ then visited and was admitted to the emergency department of Hospital B on the 18^th^. During this second stay, a sputum sample was taken and tested positive for MERS-CoV on the 20^th^ [187, 188], triggering transfer to the designated isolation treatment facility. Over a period of 10 days, the index case was seen at three different hospitals, demonstrating a key feature of “hospital shopping” that shaped the South Korean outbreak. Approximately 34 people were infected during this time [187]. In total 186 cases were generated in this outbreak, all linked through a single transmission chain to 68 M; 37 cases died [189]. In South Korea, the national health insurance system provides for relatively low cost medical care, defraying some costs by making family members responsible for a portion of the ministration of the sick, resulting in them sometimes staying for long periods in the rooms that often have more than four beds in them [24]. Other factors thought to have enabled this outbreak included unfamiliarity of local clinicians with MERS, ease with which the public can visit and be treated by tertiary hospitals, the custom of visiting sick friends and relatives in hospitals, the hierarchical nature of Korean society, crowded emergency rooms, poor IPC measures, a lack of negative pressure isolation rooms and poor inter-hospital communication of patient disease histories [24, 190–192]. All of the reported transmission occurred across three or four generations and apart from one unknown source, were all hospital-acquired [24, 120, 181, 193–195]. Few clinical details about these cases have been reported to date and detail on transmission and contact tracing is minimal. The hospitals involved were initially not identified, governmental guidance and actions produced confusing messages and there was very limited communication at all early on which resulted in unnecessary concern, distrust and a distinct economic impact [191, 196–198]. Early in the outbreak, a infected traveller, the son of an identified case in South Korea, passed through Hong Kong on his way to China where he was located, isolated and cared for in China [91, 199, 200]. No contacts became ill. The outbreak was brought under control in late July/ early August [201] after improved IPC measures were employed, strong contact tracing monitoring and quarantine, expanded laboratory testing, hospitals were better secured, specialized personnel were dispatched to manage cases and international cooperation increased [202, 203]. A review of public data showed that, as for MERS in the KSA, older age and the presence of underlying disease were significantly associated with a fatal outcome in South Korea. [40] Even though R_0_ is <1, super-spreading events facilitated by circumstances created in healthcare settings and characterized by cluster sizes over 150, such as this one, are not unexpected from MERS-CoV infection [204]. The dynamic of an outbreak depends on the R_0_ and an individual’s viral shedding patterns, contact type and frequency, hospital procedures and population structure and density [204].

In the region of Ar Riyad, including the capital city of Riyadh, a hospital based cluster began, within a single hospital, from late June 2015 [205]. By mid-September there had been approximately170 cases reported but the outbreak appeared to been brought under control in November.

## Conclusions

It became apparent early on that MERS-CoV spread relatively ineffectively from human-to-human. Despite ongoing and possibly seasonal introduction of virus to the human population via infected DCs and perhaps other animals yet to be identified, the vast majority of MERS-CoV transmission has occurred from infected to uninfected humans in close and prolonged contact through circumstances created by poor infection control in health care settings. This opportunistic virus has had its greatest impact on those with underlying diseases and such vulnerable people, sometimes suffering multiple comorbidities, have been most often associated with hospitals, creating a perfect storm of exposure, transmission and mortality. It remains unclear if this group are uniquely affected by MERS-CoV or if other respiratory virus infections, including those from HCoVs, produce a similarly serious impact. In South Korea, a single imported case created an outbreak of 185 cases and 36 deaths that had a disproportionate impact on economic performance, community behaviour and trust in government and the health care system. Household human-to human transmission occurs but is also limited. Educational programs will be essential tools for combatting the spread of MERS-CoV both within urban and regional communities and for the health care setting.

Vigilance remains important for containment since MERS-CoV is a virus with a genetic makeup that has been observed for only three years and is not stable. Among all humans reported to be infected, nearly 40 % have died. Continued laboratory testing, sequencing, analysis, timely data sharing and clear communication are essential for such vigilance to be effective. Global alignment of case definitions would further aid accurate calculation of a case fatality ratio by including subclinical case numbers. Whole genome sequencing has been used extensively to study MERS-CoV travel and variation and although it remains a tool for experts, it appears to be the best tool for the job.

MERS and SARS have some clinical similarities but they also diverge significantly [206]. Defining characteristics include the higher PFC among MERS cases (above 50 % in 2013 and currently at 30-40 %; well above the 9 % of SARS) and the higher association between fatal MERS and older males with underlying comorbidities. For the viruses, MERS-CoV has a broader tropism, grows more rapidly in vitro, more rapidly induces cytopathogenic change, triggers distinct transcriptional responses, makes use of a different receptor, induces a more proinflammatory state and has a delayed innate antiviral response compared to SARS-CoV.

There appears to be a 2-3 % prevalence of MERS-CoV in the KSA with a 5 % chance of secondary transmission within the household. There is an increased risk of infection through certain occupations at certain times and a much greater chance for spread to other humans during circumstances created by humans, which drives more effective transmission than any R_0_would predict on face value. Nonetheless, despite multiple mass gatherings that have afforded the virus many millions of opportunities to spread, there have remarkably been no reported outbreaks of MERS or MERS-CoV during or immediately after these events. There is no evidence that MERS-CoV is a virus of pandemic concern. Nonetheless, hospital settings continue to describe MERS cases and outbreaks in the Arabian Peninsula. As long as we facilitate the spread of MERS-CoV among our most vulnerable populations, the world must remain on alert for cases which may be exported more frequently when a host country with infected camel reservoirs is experiencing human clusters or outbreaks.

The MERS-CoV appears to be an enzootic virus infecting the DC URT with evidence of recent genetic recombination. It may once have had its origins among bats, but evidence is lacking and the relevance of that to today’s ongoing epidemic is academic. Thanks to quick action, the sensitive and rapid molecular diagnostic tools required to achieve rapid and sensitive detection goal have been in place and made widely available since the virus was reported in 2012. RT-PCR testing of LRT samples remains the gold standard for MERS-CoV confirmation. Serological tools continue to emerge but they are in need of further validation using samples from mild and asymptomatic infections and a densely sampled cohort study to follow contacts of new cases may address this need. Similarly, the important question of whether those who do shed MERS-CoV RNA for extended periods are infectious while appearing well, continues to go unanswered. It is even unclear just how many ‘asymptomatic’ infections have been described and reported correctly which in turn raises questions about the reliability of other clinical data collection to date. While the basic virology of MERS-CoV has advanced over the course of the past three years, understanding what is happening in, and the interplay between, camel, environment and human is still in its infancy.

## Additional files

Additional file 1: Figure S1. The 26 countries in which MERS-CoV has been identified and a guide as to the number of cases at each location. Local transmission in 13 countries is highlighted (blue star) as are countries with DCs that contain antibodies reactive with MERS-CoV, viral RNA or infectious virus (camel icon). Correct as of the 29^th^August, 2015. Adapted and reprinted from Mackay IM, Arden KE. Middle East respiratory syndrome: An emerging coronavirus infection tracked by the crowd. Virus Res 2015 Vol 202:60–88 with permission from Elsevier [5]. (EPS 41077 kb) Additional file 2: Figure S2. MERS-CoV detections, age and sex pyramids. A) All MERS-CoV detections worldwide and B) those with fatal outcomes; C) The distribution of detections limited to the KSA cases only and D) those with a fatal outcome; E) The distribution resulting from the Jeddah-2014 outbreak, arbitrarily defined as spanning from the week beginning 17^th^ March 2014 and ending in the week beginning 7th July 2014 and F) those with a fatal outcome; G) the distribution resulting from the South Korean-2015 outbreak and H) those with a fatal outcome; I) the distribution during the Riyadh-2015 outbreak and J) those with a fatal outcome; data are based on laboratory confirmed cases collated into the author’s curated line list as at 4^th^ September 2015. Note the changed x-axis scale between A-D and E-J. Sources of these public data include the WHO, Ministries of Health and FluTrackers [206–208]. Earlier and subsequent versions of this chart are maintained on a personal blog [209]. (EPS 6702 kb)

## Abbreviations

AdV: adenovirus
BCoV: bovine coronavirus
CoV: coronavirus
DC: dromedary camel
DPP4: dipeptidyl peptidase 4
ELISA: enzyme linked immunosorbent assay
EV: enterovirus
HCoV: human coronavirus
HCW: healthcare worker
HHV: human herpesvirus
HRV: human rhinovirus
IFA: immunofluorescent assay
IFV: influenza virus
Ig: immunoglobulin
IPC: infection prevention and control
KSA: Kingdom of Saudi Arabia
LRT: lower respiratory tract
MERS: Middle East respiratory syndrome
MNT: microneutralization
MPV: human metapneumovirus
nCoV: novel coronavirus
NT: neutralization
ORF: open reading frame
PCR: polymerase chain reaction
PIV: parainfluenza virus
PPE: personal protective equipment
ppNT: pseudo particle neutralization
R_0_: basic reproduction number
RBD: receptor binding domain
RH: relative humidity
RNA: ribonucleic acid
RSV: respiratory syncytial virus
RT-rtPCR: reverse transcriptase real-time polymerase chain reaction
SARS: Severe acute respiratory syndrome
TCID_50_: 50 % tissue culture infectious dose
UAE: United Arab Emirates
URT: upper respiratory tract
US: United States of America
WHO: World Health Organization
